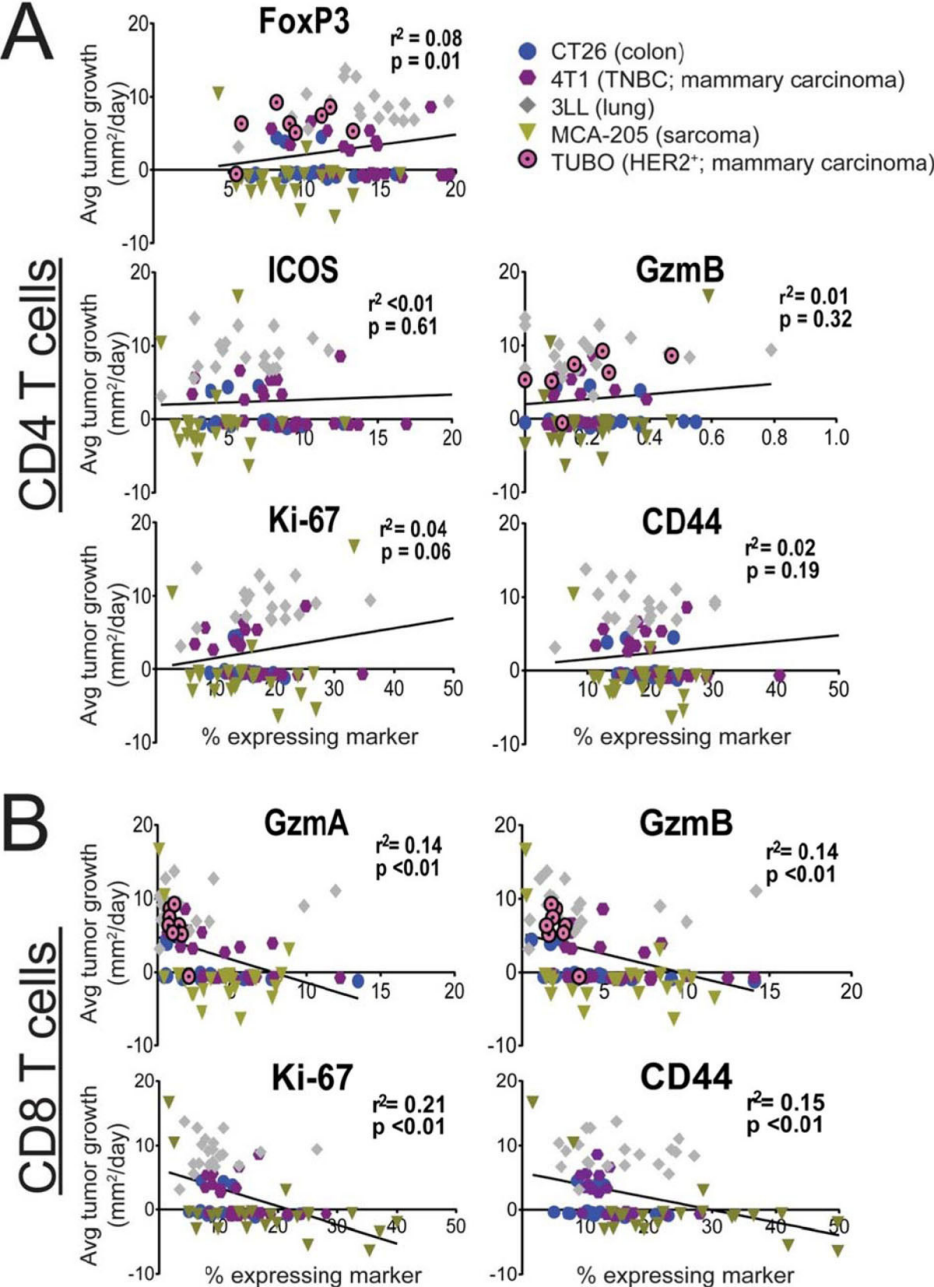# Murine peripheral blood prognostic biomarkers for tumor survival following combination aCTLA-4 and aPD-1 treatment

**DOI:** 10.1186/2051-1426-3-S2-P91

**Published:** 2015-11-04

**Authors:** Ian Hilgart-Martiszus, Michael McNamara, William Redmond

**Affiliations:** 1 Earle A Chiles Research Institute, Portland, OR, USA; 2 Providence Cancer Center, Portland, OR, USA

## Background

Immune checkpoint inhibitors, particularly those targeting CTLA-4 and PD-1, are transforming the way cancer is treated. However, these therapies do not benefit all patients and frequently cause significant immune-related adverse events. Therefore, prognostic biomarkers that identify positively-responding patients, early in the course of therapy, are essential for guiding treatment decisions and improving patient outcomes.

## Methods

In this study, we present evidence that shortly after initiating combination PD-1/CTLA-4 blockade, there is a transient increase in the frequency of pro-inflammatory and cytotoxic lymphocytes in peripheral blood, and the dynamics of this shift correlate with survival outcomes in multiple murine models.

## Results

Specifically, we observed that 1) the relative frequency of cytotoxic CD8 T cells among peripheral lymphocytes and 2) the pro-inflammatory capacity of peripheral lymphocytes are both predictive for outcomes at an early time point. Surprisingly, robust correlations between peripheral lymphocyte markers and outcomes were limited to CD8 T cell populations. In general, the expression of potential biomarkers on peripheral CD4 T cells, including ICOS and FoxP3, were poorly correlated with outcomes in this study.

## Conclusions

Overall, these findings suggest that elements of the near-term peripheral immune response to dual anti-PD-1/anti-CTLA-4 therapy associated with cytotoxic lymphocyte function may provide unique prognostic biomarkers for therapeutic outcomes.

**Figure 1:**